# Study of the Size and Shape of Synapses in the Juvenile Rat Somatosensory Cortex with 3D Electron Microscopy

**DOI:** 10.1523/ENEURO.0377-17.2017

**Published:** 2018-01-30

**Authors:** Andrea Santuy, José-Rodrigo Rodríguez, Javier DeFelipe, Angel Merchán-Pérez

**Affiliations:** 1 Laboratorio Cajal de Circuitos Corticales, Centro de Tecnología Biomédica, Universidad Politécnica de Madrid. Pozuelo de Alarcón, Madrid 28223, Spain; 2 Instituto Cajal, Consejo Superior de Investigaciones Científicas, Madrid 28002, Spain; 3 Departamento de Arquitectura y Tecnología de Sistemas Informáticos, Universidad Politécnica de Madrid, Madrid 28660, Spain

**Keywords:** cortex, semiautomated electron microscopy, synaptic size, somatosensory, synapses, focused ion beam milling and scanning electron microscopy (FIB/SEM)

## Abstract

Changes in the size of the synaptic junction are thought to have significant functional consequences. We used focused ion beam milling and scanning electron microscopy (FIB/SEM) to obtain stacks of serial sections from the six layers of the rat somatosensory cortex. We have segmented in 3D a large number of synapses (*n* = 6891) to analyze the size and shape of excitatory (asymmetric) and inhibitory (symmetric) synapses, using dedicated software. This study provided three main findings. Firstly, the mean synaptic sizes were smaller for asymmetric than for symmetric synapses in all cortical layers. In all cases, synaptic junction sizes followed a log-normal distribution. Secondly, most cortical synapses had disc-shaped postsynaptic densities (PSDs; 93%). A few were perforated (4.5%), while a smaller proportion (2.5%) showed a tortuous horseshoe-shaped perimeter. Thirdly, the curvature was larger for symmetric than for asymmetric synapses in all layers. However, there was no correlation between synaptic area and curvature.

## Significance Statement

The size of synapses correlates with functional aspects such as the probability of neurotransmitter release or the number of postsynaptic receptors. The data obtained in the present study is based on the analysis of thousands synaptic junctions, that have been imaged and segmented in 3D with semiautomated electron microcopy and image analysis methods, providing a robust set of morphologic data. Since currently-available 3D quantitative data are scarce and mainly based on individual cases, the present results in conjunction with other crucial microanatomical data, such as the number and distribution of different types of synapses and the identification of postsynaptic targets in different cortical layers, will help to better understand the structure of microcircuits and to build realistic cortical models.

## Introduction

There are two main types of chemical synapses in the cerebral cortex that can be identified at the electron microscope level based on morphologic criteria: asymmetric synapses (AS), that have a thickened postsynaptic density (PSD) and are generally excitatory (glutamatergic), and symmetric synapses (SS), that have a thinner PSD and are inhibitory (GABAergic; Houser et al., 1984; Peters et al., 1991; Ascoli et al., 2008). In the cerebral cortex, the vast majority of synapses are established in the neuropil which represents 90−98% of the volume of the gray matter (Alonso-Nanclares et al., 2008). In the neuropil, which is composed of dendrites, axons and glial processes, most cortical synapses are excitatory (80−90%) and originate from spiny neurons and extrinsic cortical afferents. Inhibitory synapses are less numerous (∼10−20%) and mainly originate from local interneurons (Feldman, 1984; Beaulieu and Colonnier, 1985; Schüz and Palm, 1989; White and Keller, 1989; DeFelipe and Fariñas, 1992; DeFelipe et al., 2002; White, 2007; Silberberg, 2008).

Synapses are dynamic structures than can undergo modifications due to variations in their activity patterns; they are continuously remodeled and replaced as part of the normal maintenance of the brain (Fauth and Tetzlaff, 2016; Lisman, 2017). This is important because the size of the active zone (AZ) is proportional to the number of docked synaptic vesicles and to the probability of neurotransmitter release (Schikorski and Stevens, 1997; Branco et al., 2010; Matz et al., 2010; Holderith et al., 2012), and the PSD area is proportional to the number of postsynaptic receptors (Nusser et al., 1998; Kharazia and Weinberg, 1999; Takumi et al., 1999; Ganeshina et al., 2004a,b; Tarusawa et al., 2009). Thus, changes in the surface areas of the AZ and PSD have significant functional consequences. However, measuring the size of a synapse is not an easy task and different approaches have been used to overcome this difficulty. The cross-sectional length of the PSDs in single photomicrographs obtained by transmission electron microscopy (TEM) gives a rough estimate of synaptic sizes (DeFelipe et al., 1999). Better estimates can be obtained from TEM serial sections, measuring the maximum width of the PSDs (Tarusawa et al., 2009) or the PSD surface area (Toni et al., 2001). However, serial sectioning is a time-consuming and technically demanding task. Consequently, data on synaptic sizes are either relatively inaccurate or based on relatively scant data. Recently, new electron microscopy techniques have been developed that allow us to obtain long series of sections in an automated way (Denk and Horstmann, 2004; Smith, 2007; Knott et al., 2008; Merchán-Pérez et al., 2009; Helmstaedter, 2013; Morgan and Lichtman, 2013). For example, using focused ion beam milling and scanning electron microscopy (FIB/SEM), large numbers of synaptic junctions can be 3D segmented from serial sections (Morales et al., 2011). Simple measurements, such as the Feret’s diameter (the diameter of the smallest sphere circumscribing the 3D object) can be obtained (Anton-Sanchez et al., 2014; Merchán-Pérez et al., 2014). The main advantage of Feret’s diameter is its simplicity, although it provides no information about shape. A more accurate method has been developed to estimate the size and shape of synapses. Since the AZ and the PSD are located face to face and their surface areas are very similar (Schikorski and Stevens, 1997, 1999) they can be represented by a single surface, the synaptic apposition surface (SAS), that can be automatically extracted with dedicated software (Morales et al., 2013). The SAS provides not only quantitative information, but also qualitative visual information about the shape of the synaptic junction, such as curvature, perimeter tortuosity or the presence of perforations.

In this work, we have studied the size and shape of AS and SS on spines and dendritic shafts in the neuropil of all cortical layers of the somatosensory cortex. We used a large database of synaptic junctions that were fully segmented in 3D (*n* = 6891) from Wistar rats at postnatal day 14. We extracted and measured the SAS of these synapses using the method developed by Morales et al. (2013). This experimental animal, at this age, was selected since we intended to integrate these data with other anatomic, molecular, and physiologic data that have already been collected from the same cortical region. The final goal is to obtain accurate quantitative data that help to create a detailed, biologically accurate model of circuitry for all layers in the primary somatosensory cortex, within the framework of the Blue Brain Project (Markram et al., 2015).

## Materials and Methods

### Animals and tissue preparation

Three male Wistar rats sacrificed on postnatal day 14 were used for this study. Animals were administered a lethal intraperitoneal injection of sodium pentobarbital (40 mg/kg) and were intracardially perfused with 2% paraformaldehyde and 2.5% glutaraldehyde in 0.1 M phosphate buffer (PB). The brain was then extracted from the skull and processed for electron microscopy according to a previously described protocol (Merchán-Pérez et al., 2009). Briefly, the brains were extracted from the skull and post-fixed at 4°C overnight in the same solution. Vibratome sections were obtained (150 µm thick). Sections containing the primary somatosensory cortex (hindlimb representation) were selected with the help of an atlas (Paxinos and Watson, 2007). The sections were then osmicated for 1 h at room temperature in PB with 1% OsO_4_, 7% glucose and 0.02 M CaCl_2_. After washing in PB, the sections were stained en bloc for 30 min with 1% uranyl acetate in 50% ethanol at 37°C, and were then flat-embedded in Araldite. These tissue samples have been used previously to describe the proportions and densities of AS and SS on spines and dendritic shafts across all cortical layers, as well as the occurrence of single or multiple synapses on the same spine (Santuy et al., 2018).

All animals were handled in accordance with the guidelines for animal research set out in the European Community Directive 2010/63/EU, and all procedures were approved by the local ethics committee of the Spanish National Research Council (CSIC).

### 3D electron microscopy

3D brain tissue samples of the somatosensory cortex (hindlimb representation) were obtained using combined FIB/SEM. We used a Crossbeam Neon40 EsB electron microscope with a field emission SEM column and a Gallium FIB (Carl Zeiss NTS GmbH). To select the exact location to be imaged and to identify the cortical layers, we obtained semithin sections (2 μm thick) from the block surface and stained them with toluidine blue. These sections were then photographed with a light microscope. The last of these light microscope images (corresponding to the section immediately adjacent to the block face) was then collated with SEM photographs of the block face. A gallium ion beam was used to mill the sample, removing thin layers of material on a nanometer scale. After removing each slice (20 nm thick), the milling process was paused, and the freshly exposed surface was imaged with a 1.8-kV acceleration potential using an in-column energy selective backscattered electron detector. The milling and imaging processes were sequentially repeated in a fully automated way, and long series of images were acquired, thus obtaining a stack of images that represented a 3D sample of the tissue (Merchán-Pérez et al., 2009). Twenty-nine different stacks of images of the neuropil in the six layers of the somatosensory cortex were obtained (three samples from Layer I, four from Layer II, 10 from Layer III, five from Layer IV, three from Layer V, and four from Layer VI). All these stacks were used previously for the study of the density and 3D distribution of synapses (Anton-Sanchez et al., 2014; Merchán-Pérez et al., 2014), as well as for the quantitative estimation of the subcellular location of synapses on spines and dendritic shafts (Santuy et al., 2018). This study was performed in the neuropil, so we used stacks of images that did not contain cell somata or blood vessels. Image resolution in the xy plane ranged from 3.7–4.5 nm/pixel. Resolution in the *z* axis (section thickness) was 20 nm. With these resolution parameters, we obtained images of 2048 × 1536 pixels, so the field of view was 7.56 × 5.68 µm at 3.7 nm/pixel. Noise reduction was performed by line averaging, and the acquisition time per image was approximately four minutes. Although the resolution of FIB/SEM images can be increased, we chose these parameters as a compromise solution to obtain a large enough field of view where synaptic junctions could still be clearly identified, in a period of time that allowed us to acquire between 189 and 363 serial sections per stack (mean: 254.66; total: 7385 sections).

### Extraction of the synaptic apposition surface

Synaptic junctions within these volumes were visualized and automatically segmented in 3D with EspINA software (Morales et al., 2011). The segmentation algorithm makes use of the fact that presynaptic density and PSD appear as dark, electron-dense structures under the electron microscope. It requires a Gaussian blur filter preprocessing step to eliminate noisy pixels, followed by a gray-level threshold to extract all the voxels that fit the gray levels of the synaptic junction. In this way, the resulting 3D segmentation includes both the presynaptic density and PSD. Since the presynaptic density and PSD are located face to face, their surface areas are very similar (correlation coefficients over 0.97; Schikorski and Stevens, 1997, 1999). Thus, they can be simplified to a single surface and represented as the surface of apposition between the presynaptic density and the PSD. This surface can be extracted from the 3D segmented synaptic junction (Morales et al., 2013). For the sake of clarity, we will refer to this surface as the synaptic apposition surface (SAS). EspINA was used to visualize the SAS in 3D and the possible presence of perforations or deep indentations in the perimeter were recorded. EspINA was also used to measure SAS areas and perimeters. Since the SAS adapts to the curvature of the synaptic junction, we have also measured its curvature as one minus the ratio between the projected area of the SAS and the area of the SAS. This measure would equal 0 in a totally flat SAS, and the value would increase up to a maximum of 1 as the SAS curvature increases. All measurements have been corrected for tissue shrinkage due to processing for electron microscopy (Merchán-Pérez et al., 2009). Correction factors for volume, surface and linear measurements were 0.73, 0.81, and 0.90, respectively.

### Statistical analysis

To study whether there were significant differences we performed multiple mean comparison tests on the 29 samples of the six cortical layers. Since the necessary assumptions for ANOVA were not satisfied (the normality and homoscedasticity criteria were not met), we used the Mann–Whitney test for pair-wise comparisons. χ^2^ Tests were used for contingency tables. Linear regression was used to find correlations. SPSS 22.0 (IBM Corp.) and Easyfit Professional 5.5 (MathWave Technologies) were used.

## Results

### Synaptic junction areas and perimeters

**Figure 1.**
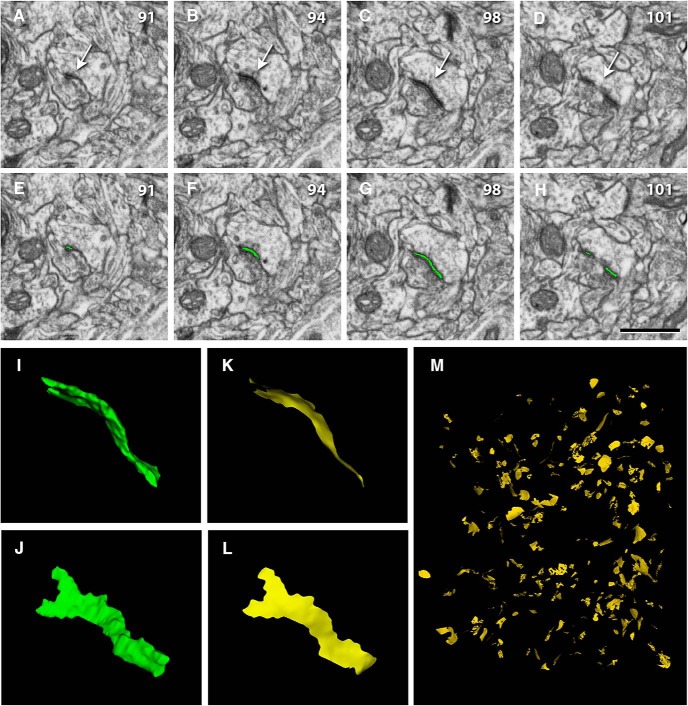
Identification, segmentation, and extraction of the SAS of a synaptic junction from serial images obtained with combined FIB/SEM. ***A–D***, Sections 91, 94, 98, and 101 from a stack of serial sections obtained with FIB/SEM from the rat somatosensory cortex. Identification of an AS whose prominent PSD is clearly visible (arrow). Note that the identification was not based on single images but on the examination of the full sequence of images where the synapse was visible (numbers in the top-right corner of each frame correspond to section number; each individual section was 20 nm thick). ***E–H***, Segmentation of the synaptic junction (green) with EspINA software. ***I***, ***J***, The resulting 3D object representing the synaptic junction (green) visualized from two different angles. ***K***, ***L***, The SAS (yellow) that has been extracted from the 3D synaptic junction shown in ***I***, ***J***. ***M***, Panoramic view of all the SAS extracted from a whole stack of images. Scale bar: 1 µm (***A–H***).

In our samples, we found 7569 synaptic junctions. We discarded 678 (8.96%) because they were truncated by the edges of the field of view. Thus, we finally analyzed 6891 synapses whose synaptic junctions were complete, so their SAS could be extracted (Fig. 1). Of these, 6259 (90.83%) were AS and 632 were SS (9.17%).

**Figure 2.**
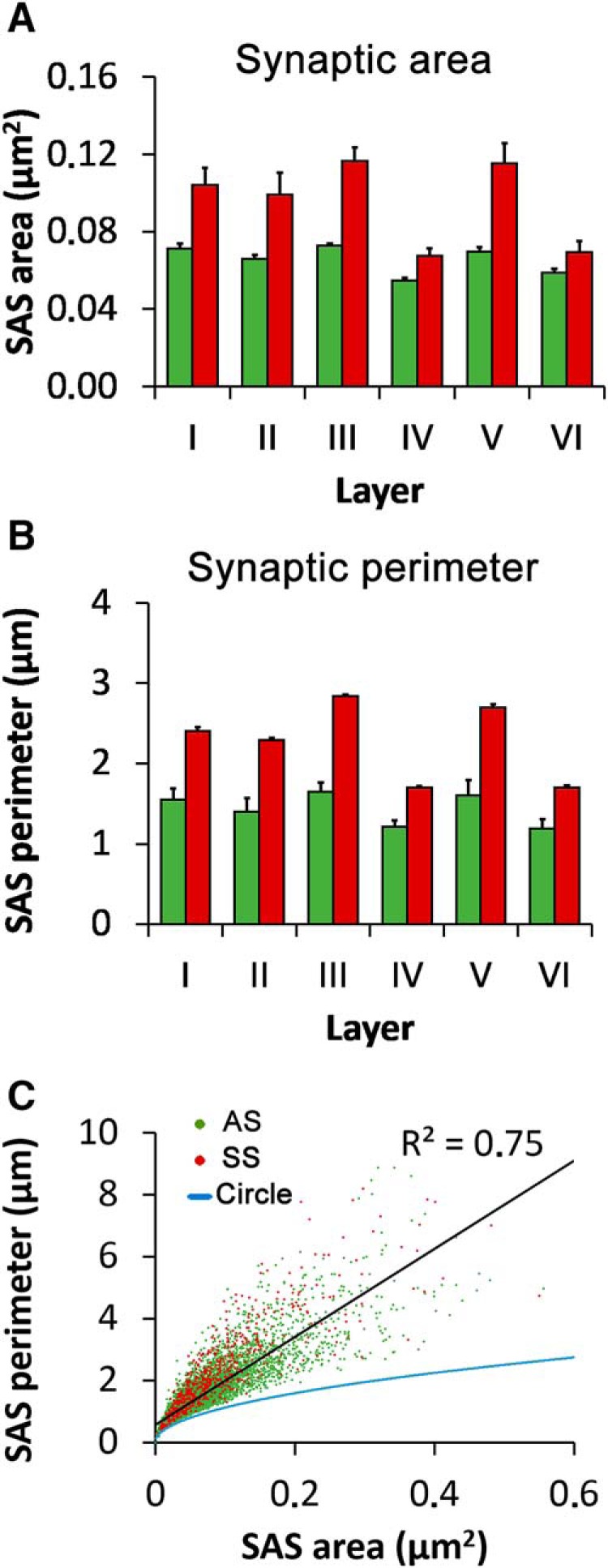
Size and perimeter of synaptic junctions (mean ± SEM). ***A***, Surface area of SAS of AS (green bars) and SS (red bars) in the six cortical layers. AS were smaller than SS in all layers (MW tests, *p* < 0.001 in Layers I–V; *p* = 0.026 in Layer VI). For both AS and SS, the largest SAS were found in Layer III and the smallest were found in Layer IV. ***B***, Perimeters of SAS of AS (green bars) and SS (red bars) in the six cortical layers. Perimeters of SAS showed similar differences to SAS areas. ***C***, Scatter plot showing the relationship between SAS areas and perimeters. AS are represented as green dots and SS as red dots. The blue trace indicates the perimeter/area relation of a circle, as a reference. There is a strong correlation between SAS area and perimeter (*R*^2^ = 0.75 for AS and SS pooled together, black trace). If we compare the perimeter/area relation of a circle (blue trace) with the SAS perimeter/area plot of SAS, it is clear that SAS perimeters grow faster than the perimeter of a circle, indicating that SAS perimeters tend to be more tortuous as SAS area increases.

SAS areas ranged from 909.23 to 556,393.19 nm^2^ for AS, and from 3,388.21 to 631,774.04 nm^2^ for SS. Mean SAS areas were smaller for AS than for SS in all cortical layers (Table 1; Fig. 2*A*), and these differences were statistically significant in all cases (MW tests, *p* < 0.001 in Layers I–V and *p* = 0.026 in Layer VI). For AS, the largest mean SAS areas were found in Layer III (mean = 72,729.58 nm^2^), and the differences between this layer and all other layers were statistically significant (MW, *p* ≤ 0.023). The smallest mean SAS areas of AS were found in Layer IV (mean = 54,770.81 nm^2^), and the differences between this layer and Layers I, III, and V were statistically significant (MW, *p* ≤ 0.001). For SS, the largest mean SAS areas were also found in Layer III (mean = 116,703.43 nm^2^; differences were statistically significant between this layer and Layers II, IV, and VI; MW, *p* ≤ 0.002). The smallest mean SAS areas of SS were found in Layer IV (mean = 68,355.35 nm^2^) and the differences were statistically significant between this layer and all other layers except Layer VI (MW, *p* ≤ 0.031).

**Table 1.** Mean SAS area (nm^2^ ± SEM), number of synaptic SAS analyzed (*n*), and the location (µ), and scale (σ) of the best-fit log-normal distributions in the six cortical layers

	AS				SS			
	Mean SAS area ± SEM (nm^2^)	*n*	μ	σ	Mean SAS area ± SEM (nm^2^)	*n*	μ	σ
Layer I	70,834.87 ± 2649.41	594	10.81	0.88	104,309.94 ± 8741.68	77	11.29	0.78
Layer II	58,987.37 ± 1788.36	992	10.72	0.83	87,757.53 ± 8875.47	64	11.14	0.72
Layer III	72,729.58 ± 1209.78	2212	10.93	0.74	116,703.42 ± 6735.43	185	11.42	0.72
Layer IV	54,770.81 ± 1245.29	1200	10.65	0.73	68,355.35 ± 3896.95	172	10.88	0.73
Layer V	69,682.16 ± 2210.41	684	10.85	0.81	113,353.40 ± 10086.08	62	11.41	0.69
Layer VI	58,668.28 ± 2050.82	577	10.73	0.70	69,382.12 ± 5695.64	72	10.90	0.76
Layers I–VI	65,299.31 ± 697.16	6259	10.84	0.79	93,384.53 ± 3001.23	632	11.17	0.78

Unweighted means for Layers I–VI are also given.

We also measured the perimeters of the SAS (Table 2; Fig. 2*B*). For each individual layer, mean perimeters were always larger for SS than for AS (Fig. 2*B*; Table 2). As expected, there was a strong correlation between SAS area and perimeter (*R*^2^ = 0.75 for all synapses; *R*^2^ = 0.75 for AS; *R*^2^ = 0.72 for SS; Fig. 2*C*). It is also interesting to note that the larger the SAS area, the more tortuous its perimeter. This can be seen in Figure 2*C*, which shows that the SAS perimeter tends to grow faster than the perimeter of a circle.

**Table 2.** Mean SAS perimeter (nm ± SEM), number of synapses analyzed (*n*), and the location (µ), and scale (σ) of the log-normal distributions of SAS perimeters in the six cortical layers

	AS				SS			
	Mean SAS perimeter ± SEM (nm)	*n*	μ	σ	Mean SAS perimeter ± SEM (nm)	*n*	μ	σ
Layer I	1,538.03 ± 42.13	594	7.16	0.60	2,405.01 ± 142.87	77	7.65	0.55
Layer II	1,365.80 ± 29.46	992	7.06	0.55	2,141.62 ± 130.11	64	7.55	0.52
Layer III	1,638.51 ± 19.64	2212	7.27	0.59	2,838.22 ± 118.19	185	7.81	0.54
Layer IV	1,221.79 ± 18.70	1200	6.99	0.47	1,704.83 ± 77.28	172	7.29	0.54
Layer V	1,602.44 ± 38.50	684	7.22	0.56	2,736.41 ± 186.46	62	7.77	0.50
Layer VI	1,191.59 ± 25.83	577	6.97	0.45	1,697.00 ± 112.65	72	7.29	0.55
Layers I–VI	1,460.71 ± 11.29	6259	7.14	0.54	2,266.44 ± 54.61	632	7.56	0.59

Unweighted means for Layers I–VI are also shown.

**Figure 3.**
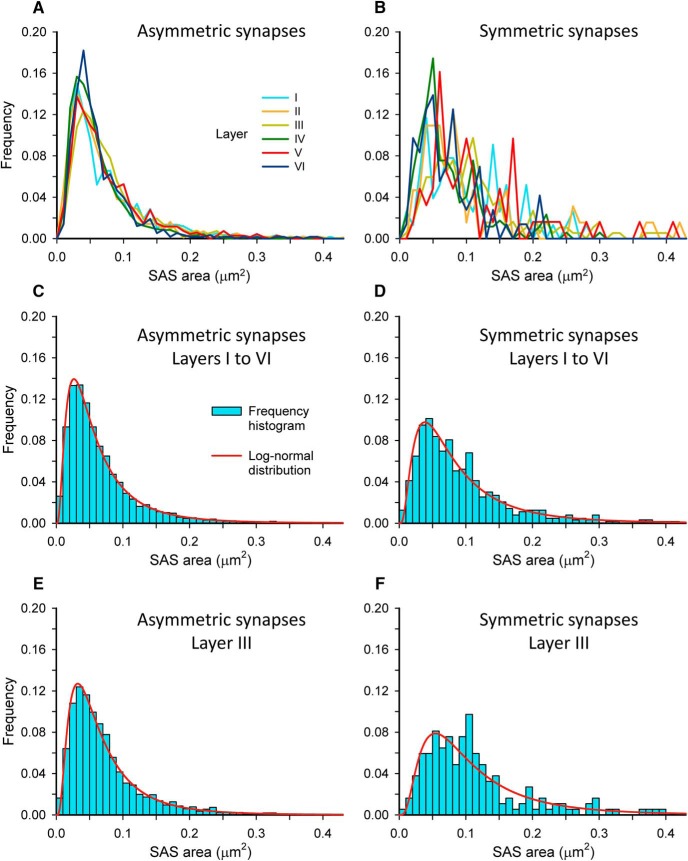
Frequency histograms of SAS areas and their corresponding best-fit probability density functions. Frequency histograms of SAS areas in the six cortical layers are represented for AS and SS in ***A***, ***B***, respectively. Histograms for AS from different layers had similar shapes and overlapped greatly, while histograms for SS were more irregular. AS and SS from all layers have been pooled together to build the frequency histograms (blue bars) represented in ***C***, ***D***. The best-fit distributions representing the theoretical probability density functions (red traces) have been represented with their corresponding frequency histograms. As an example, for an individual layer, histograms and best-fit distributions for AS and SS from Layer III have been represented in ***E***, ***F***. The best-fit probability function was a log-normal distribution in all cases. Curve fitting was always better for AS (***C***, ***E***) than for SS (***D***, ***F***), probably because of the smaller sample size of SS (Table 1). The parameters µ and σ of the log-normal curves are shown in Table 1.

To further characterize the size distribution of SAS, we plotted the frequency histograms of SAS areas for each individual layer and for all layers as a whole. For AS, frequency histograms had similar shapes in all layers, with a tail to the right, and they overlapped greatly (Fig. 3*A*). For SS, more irregular-shaped histograms were obtained for individual layers, probably due to the smaller number of synaptic junctions that were analyzed per layer (Fig. 3*B*; Table 1). We then performed goodness-of-fit tests to find the theoretical probability density functions that best fitted the empirical distributions of SAS areas in each layer and in all layers pooled together. We found that the best fit corresponded to log-normal distributions in all cases (Table 1; Fig. 3). These log-normal distributions, with some variations in the location (µ) and scale (σ) parameters (Table 1), were found in all layers for both AS and SS, although the fit was better for AS than for SS, probably due to the smaller number of SS analyzed (Fig. 3). The best-fit probability density functions for SAS perimeters were also log-normal distributions (Table 2).

### Size of synaptic junctions on dendritic spines and shafts

**Figure 4.**
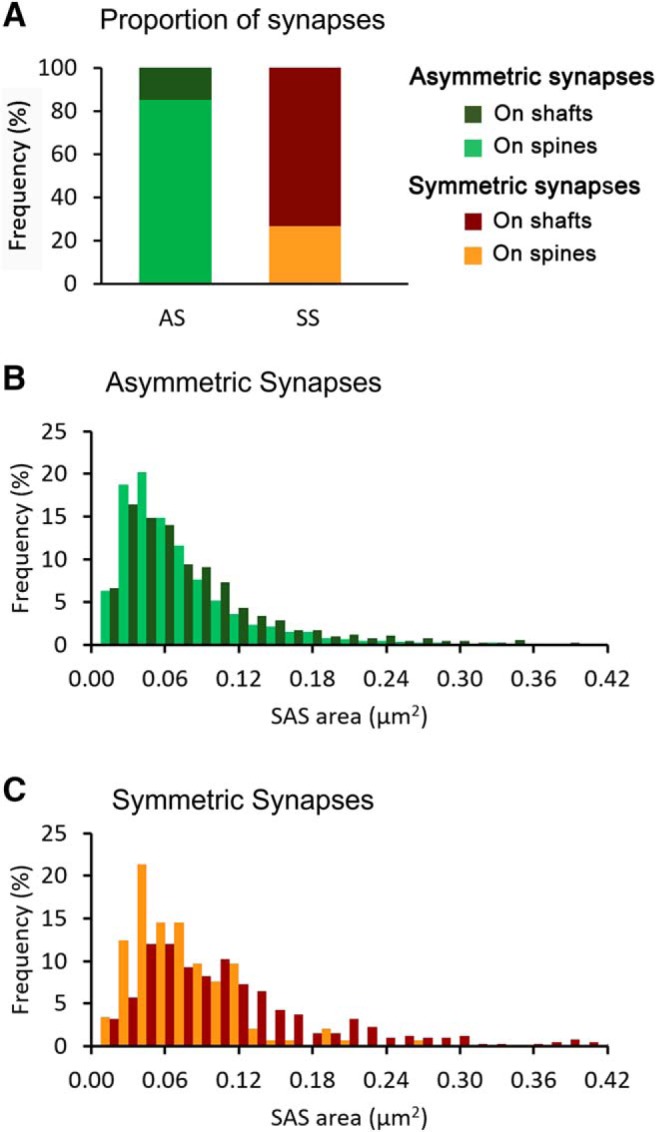
SAS areas of synapses on dendritic spines and shafts. ***A***, Proportions of AS and SS on dendritic spines and shafts. ***B***, Frequency histograms of SAS areas of AS on dendritic spines (light green) and on dendritic shafts (dark green). ***C***, Frequency histograms of SAS areas of SS on dendritic spines (orange) and on dendritic shafts (dark orange). Frequencies in ***B***, ***C*** have been normalized for each individual category.

We also determined whether the postsynaptic element where the synapses were established (dendritic spines or shafts) was associated with differences in the size of PSDs. Unambiguous identification of spines required the dendritic spine to be visually traced to the parent dendrite within the 3D stack of serial sections. Similarly, dendritic shafts needed to be followed inside the stack until they could be clearly identified. For this analysis, we studied 6000 synapses whose postsynaptic targets were successfully identified. We found that the mean SAS area of synapses located on dendritic shafts (88,795.98 nm^2^ ± SEM = 2210.16) was larger than the mean SAS area of those located on necks (57,879.38 nm^2^ ± SEM = 3998.65) and spine heads (65,164.05 nm^2^ ± SEM = 797.26; MW tests, *p* < 0.001). This difference could be due to the fact that SS, which are larger than AS, were predominantly located on dendritic shafts (in this sample, 73.39% of SS were located on shafts, while only 14.61% of AS were located on shafts). To rule out this possibility, we analyzed AS and SS independently. We found that the mean SAS area of AS located on shafts (78,255.07 nm^2^ ± SEM = 2413.73) was larger than those located on dendritic spines heads (65,196.81 nm^2^ ± SEM = 811.93; MW test, *p* < 0.001) and the ones on spines heads were larger than those on necks (52,326.09 nm^2^ ± SEM = 4394.36; MW test, *p* = 0.04). Similarly, the mean SAS areas of SS were larger on shafts (109,798.76 nm^2^ ± SEM = 4358.62) compared to dendritic spine heads (63,808.43 nm^2^ ± SEM = 3592.90) and necks (74,697.93 nm^2^ ± SEM = 8576.53; MW tests, *p* < 0.001), although the difference between SS on spine heads and necks was not statistically significant (MW tests, *p* = 0.33). Therefore, synapses located on dendritic shafts were larger than those located on dendritic spines, both for AS and SS. When single cortical layers were analyzed, we also found that the mean SAS area of synapses established on dendritic shafts was always larger than SAS areas of synapses on dendritic spines. Despite the differences in the mean SAS areas mentioned above, the frequency histograms of SAS areas of AS and SS on dendritic shafts and spines greatly overlapped, as shown in Figure 4.

### The shape of synaptic junctions

**Figure 5.**
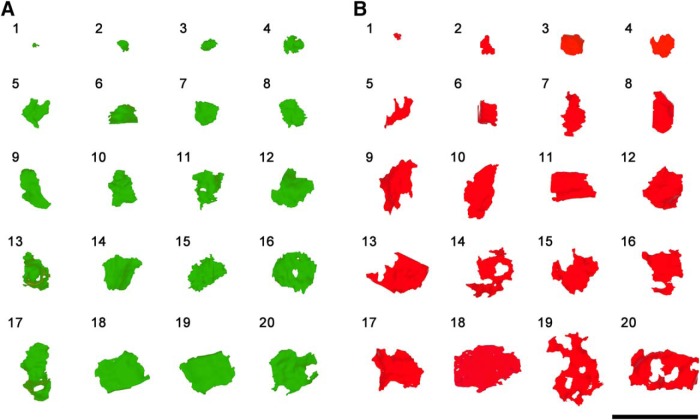
Representative sample of SAS of AS and SS. ***A***, SAS of AS (green) were distributed into 20 bins of equal size. An example within each bin has been represented here. ***B***, SAS of SS (red) that were distributed and selected as in ***A***. Scale bar: 1 µm.

The shape of synaptic junctions was very variable (Fig. 5) but can be categorized into three main types. Most cortical synapses had disk-shaped, macular PSDs (93%). A small percentage had perforations, with one or more holes in the PSD (4.5%), while an even smaller proportion (2.5%) had a tortuous horseshoe-shaped perimeter with an indentation. Macular and perforated synapses followed the previously described 9:1 proportion between AS and SS, but in the case of horseshoe-shaped PSDs, this proportion was 8:2, indicating that horseshoe-shaped synaptic junctions were relatively more frequent among SS than among AS (χ^2^, *p* < 0.001).

**Figure 6.**
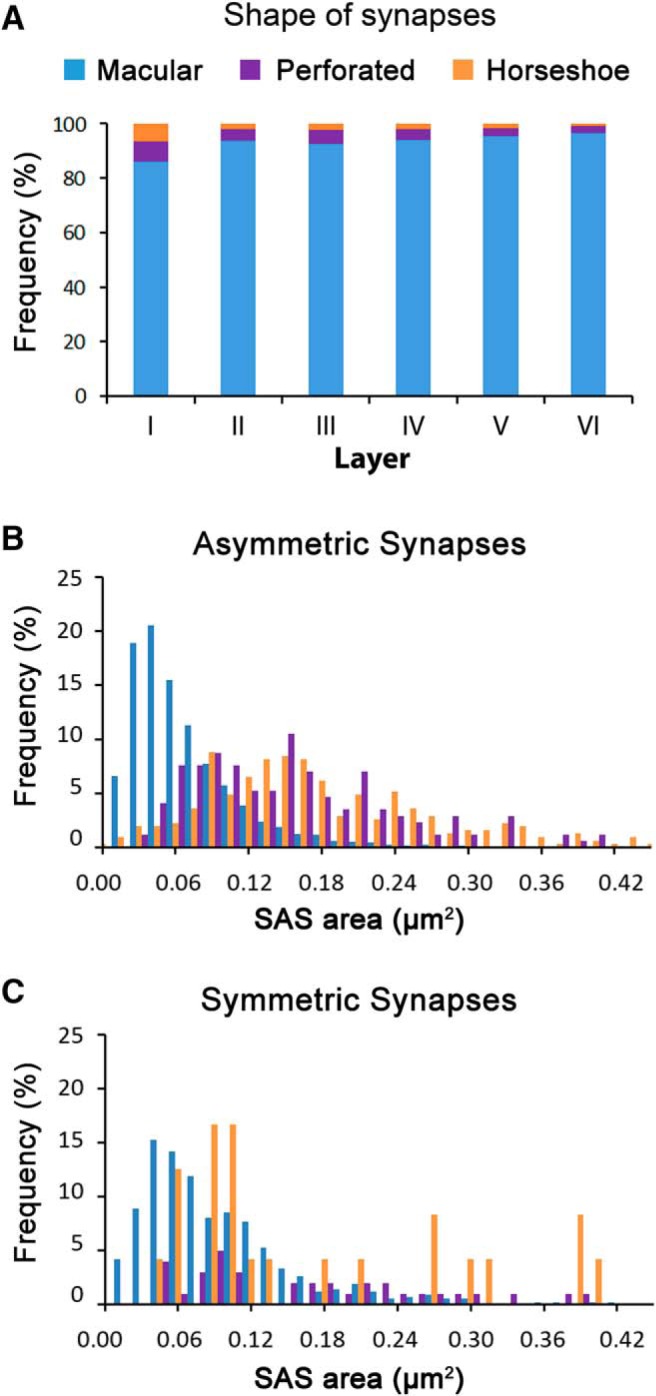
Distribution of synapses of different shapes. ***A***, Proportion of macular (blue), perforated (purple), and horseshoe-shaped (orange) synapses in the six layers of the cortex. Layer I shows a higher proportion of perforated and horseshoe synapses when compared to Layers II–VI (χ^2^, *p* < 0.001). ***B***, Frequency histograms of the SAS area of macular, perforated, and horseshoe-shaped AS. ***C***, Frequency histograms of the SAS area of macular, perforated, and horseshoe-shaped SS. Frequencies in ***B***, ***C*** have been normalized for each individual category.

The mean SAS area of macular synapses was smaller (mean ± SEM = 61,737.72 nm^2^ ± 606.10) than horseshoe-shaped synapses (148,469.66 nm^2^ ± 6321.63; MW test, *p* < 0.001) and the mean SAS area of horseshoe-shaped synaptic junctions was smaller than that of perforated synapses (176,710.07 nm^2^ ± 5875.00; MW test, *p* = 0.005; Table 3). Despite the differences in the mean SAS areas, perforated and horseshoe-shaped synaptic junctions were intermingled with the predominant macular synaptic junctions (Figs. 5, 6). The perimeter of macular synapses was shorter (1,423.96 nm ± 10.22) than horseshoe-shaped synapses (3,124.70 nm ± 107.62; MW test, *p* < 0.001) and perforated synapses (3,106.10 nm ± 87.94; MW test, *p* < 0.001), while horseshoe-shaped and perforated synapses had similar perimeters (MW test, *p* = 0.59; Table 3).

**Table 3.** Area (nm^2^), perimeter (nm), and curvature (mean ± SEM) of the SAS of macular, perforated, and horseshoe-shaped synaptic junctions

Shape of synaptic junction	Type of synapse	Area of SAS (nm^2^)mean ± SEM	Perimeter (nm)mean ± SEM	Curvaturemean ± SEM
Macular	AS	59,271.15 ± 595.95	1,353.95 ± 9.55	0.07 ± 0.001
	SS	86,903.65 ± 2816.34	2,138.30 ± 51.47	0.09 ± 0.003
	AS + SS	61,737.72 ± 606.10	1,423.96 ± 10.22	0.07 ± 0.001
Perforated	AS	175,955.57 ± 5842.02	3,056.56 ± 85.68	0.10 ± 0.004
	SS	185,606.80 ± 30,594.69	3,690.38 ± 488.31	0.08 ± 0.010
	AS + SS	176,710.07 ± 5875.00	3,106.10 ± 87.94	0.10 ± 0.003
Horseshoe-shaped	AS	146,689.44 ± 6756.57	3,015.70 ± 109.07	0.11 ± 0.006
	SS	155,387.11 ± 16,435.88	3,548.22 ± 304.55	0.08 ± 0.009
	AS + SS	148,469.66 ± 6321.63	3,124.70 ± 107.62	0.11 ± 0.005

All data are given as mean ± SEM.

For all three categories (macular, perforated, and horseshoe-shaped), SS had a larger area and perimeter than AS (Table 3), although these differences were only statistically significant for macular synapses (MW test; *p* < 0.001).

The proportions of macular, horseshoe and perforated synaptic junctions were similar in Layers II–VI. However, we found that horseshoe-shaped and perforated synapses were more common in Layer I (χ^2^, *p* < 0.001; Fig. 6*A*). No preference was found in the location of macular, perforated or horseshoe-shaped synapses on spines or dendritic shafts, and this was the case for both AS (χ^2^, *p* = 0.22) and SS (χ^2^, *p* = 0.66).

**Figure 7.**
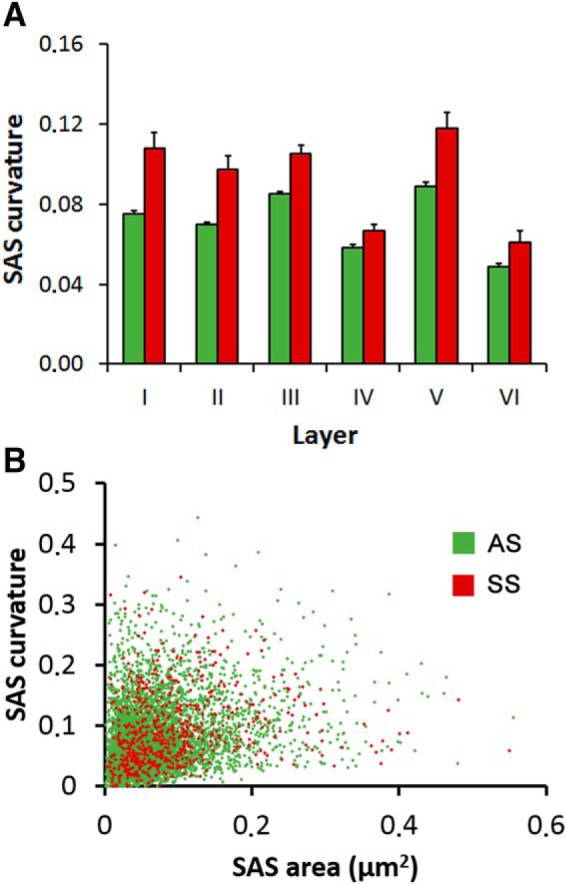
Curvature of the SAS. ***A***, SAS curvature of AS and SS in the six cortical layers (mean ± SEM). SAS curvature was larger for SS (red bars) than for AS (green bars) in all layers. For AS, statistically significant differences were found between all layers (MW tests <0.05) except between Layers I and II (MW test, *p* = 0.325) and Layers III and V (MW test, *p* = 0.14). Curvature differences between SS were found between Layers IV and VI and all the other layers (MW tests, *p* < 0.001). ***B***, Scatter plot representing the relationship between SAS curvature and area of AS (green dots) and SS (red dots). There was no correlation between SAS area and curvature for AS or SS (*R*^2^ = 0.08 for AS; *R*^2^ = 0.03 for SS).

We also measured SAS curvature by calculating one minus the ratio between the projected area of the SAS and the area of the SAS. This value would equal 0 for a totally flat SAS, and it would increase as the SAS becomes more curved or wrinkled (see Materials and Methods). Our results indicate that SAS curvature was higher for SS than for AS in all layers (MW tests, *p* ≤ 0.028; Fig. 7*A*). We made pair comparisons of AS curvature between each cortical layer and all the others and we found statistically significant differences between all layers (MW tests, *p* < 0.05) except between Layers I and II (MW test, *p* = 0.325), and Layers III and V (MW test, *p* = 0.14). For SS, statistically significant differences were found between Layers IV and VI (the ones with flattest synapses) and all the other layers (MW tests, *p* < 0.001). Macular synapses were flatter (mean ± SEM, 0.07 ± 0.001) than horseshoe-shaped (0.11 ± 0.005; MW test, *p* < 0.001) and perforated synapses (0.10 ± 0.003; MW test *p* < 0.001). Horseshoe-shaped and perforated synapses had similar curvature (MW test, *p* = 0.58; Table 3). We found no correlation between SAS area and curvature (*R*^2^ = 0.08 for AS; *R*^2^ = 0.03 for SS; Fig. 7*B*).

## Discussion

In the present study, we used a new method to estimate the size and shape of synapses that involved extracting the SAS from synaptic junctions segmented in 3D, using combined focused ion beam milling and scanning electron microscopy. This study provided three main findings. Firstly, the mean SAS areas were smaller for AS than for SS in all cortical layers and these differences were statistically significant in all cases. For both AS and SS, the largest mean SAS areas were found in Layer III and the smallest mean SAS areas were found in Layer IV. In all cases (AS and SS, in all layers), the distributions of synaptic junction size followed a skewed curve with a long tail to the right, corresponding to a log-normal distribution. Secondly, most cortical synapses had disk-shaped, macular PSDs (93%). A few were perforated, with one or more holes in the PSD (4.5%), while an even smaller proportion (2.5%) showed a tortuous horseshoe-shaped perimeter with a deep indentation. Thirdly, the SAS curvature was larger for SS than for AS in all layers. However, there was no correlation between SAS area and curvature for AS or SS.

### Methods to estimate the size of synaptic junctions

Several methods have traditionally been used to estimate the size of synaptic junctions. The simplest of these methods is to measure the cross-sectional length of synaptic junctions in TEM micrographs. This method has obvious limitations since it is based on individual 2D images where a portion of synapses cannot be fully characterized (DeFelipe et al., 1999), and it also reduces size estimation to a 1D measurement that is not equivalent to any easily interpretable geometrical measure such as the mean diameter, for example. Methods that use serial sections can yield more reliable measurements, even if only simple measurements such as the maximum width of the PSD are used (Tarusawa et al., 2009). The cross-sectional length of the PSD can also be measured in each section of the series and multiplied by section thickness and by number of sections (Arellano et al., 2007; Bopp et al., 2017; Hsu et al., 2017). Alternatively, the PSD can be reconstructed from the series of sections and its contour can be measured in 3D (Bosch et al., 2015, 2016; Rollenhagen et al., 2015; Dufour et al., 2016; Rodriguez-Moreno et al., 2017). Another measurement that has been used to estimate the size of synaptic junctions in 3D is the diameter of Feret, which is equivalent to the diameter of the smallest sphere circumscribing the reconstructed object (Merchán-Pérez et al., 2014). The Feret’s diameter is a simple and reliable measurement that can be automatically obtained at a low computational cost, and it has been shown to be useful to build models that reproduce the distribution of synapses in 3D space (Anton-Sanchez et al., 2014). However, it does not accurately describe the morphology of synapses, since it obviously oversimplifies the geometric characteristics of the measured object, and it is clear that objects with very different morphologies can have similar Feret’s diameters. Another indirect measurement of the size of the synaptic junction is the axon-spine interface (ASI), which represents the total apposition surface between the membrane of the axonal bouton and the membrane of the dendritic spine (de Vivo et al., 2017). We have used the SAS, which is equivalent to the interface between the AZ and the PSD. Therefore, although the area of the ASI and the PSD are correlated (Cheetham et al., 2014), data from de Vivo et al. (2017) are not comparable with ours, except for the fact that our measurements of the SAS are smaller than their measurements of the ASI. This is because the SAS is always inside the ASI and thus it is smaller than the ASI. Moreover, our methodology provides information on the *shape* of the PSD, as well as information about synapses established on dendritic shafts, that cannot be obtained from ASI measurements.

### SAS

In the present study, we used the SAS because it has three main advantages over the methods outlined above. First, it is extracted automatically from the previously segmented synaptic junction with no user intervention, thus avoiding any manual tracing of contours and possible associated user bias (Alonso-Nanclares et al., 2013; Morales et al., 2013). Second, despite being a surface, the SAS is also a 3D object that adapts to, and reproduces the shape and curvature of the PSD. Therefore, the SAS can be visualized in 3D to obtain qualitative information such as the presence of perforations or indentations (Fig. 5). Third, quantitative information on the surface area, perimeter and curvature can also be extracted from the SAS, so size and shape can easily be correlated. Given that the initial segmentation of synaptic junctions has been performed within 3D tissue samples using a semiautomatic method (Morales et al., 2011), and the SAS have been extracted in a fully automated way, we have been able to obtain 6891 synaptic junctions whose shape and size have been analyzed in the six cortical layers. Additionally, the postsynaptic target (dendritic spines or shafts) has been unambiguously identified in 6000 of these synaptic junctions.

### Size of synaptic junctions

The size of both types of synaptic junctions (asymmetric and symmetric) follows log-normal distributions. Despite the fact that the mean SAS area is larger for SS than for AS, their respective distributions greatly overlap (Fig. 3), so it would be impossible to distinguish AS from SS on the basis of synaptic junction size alone. It is tempting to correlate the log-normal distribution of synaptic sizes with other parameters such as synaptic strength and spike transmission probability, which also follow log-normal distributions (for review, see Buzsáki and Mizuseki, 2014). For example, the distribution of the size of unitary excitatory postsynaptic potentials (EPSP) is very similar to the distribution of the size of SAS reported here, with a skewed envelope and a long tail to the right (Song et al., 2005; Lefort et al., 2009). Moreover, the EPSP amplitude strongly correlates with the number of postsynaptic AMPA receptors and with spine head volume (Matsuzaki et al., 2001; Kasai et al., 2003; Araya, 2014), which in turn strongly correlates with PSD size (Arellano et al., 2007). Model experiments also suggest that PSD size has a strong influence on the activation of postsynaptic receptors (Montes et al., 2015). It is also interesting to note that the event-to-event variability of synaptic strength for individual synapses is largest for weaker synapses and decreases for stronger synapses (Lefort et al., 2009; Ikegaya et al., 2013). This may also be related to synaptic junction size, since the same phenomenon, a decrease in variability as synaptic size increases, has been described in model experiments (Franks et al., 2002; Montes et al., 2015). This suggests that large synapses have a higher number of receptors and are not only stronger, but also have a more homogeneous and reliable response. However, it is important to note that the amplitude of the EPSP also depends on the geometry of postsynaptic dendrites (Major et al., 2013; Eyal et al., 2014), as well as on the morphology of dendritic spines (Gulledge et al., 2012; Araya, 2014). Another important source of variability is the number of postsynaptic receptor molecules in individual synapses. For example, it has been shown in the hippocampus that the number of AMPA receptors as a function of synaptic size has different slopes in the synapses established between Shaffer collaterals and CA1 dendritic spines and in the synapses between mossy fibers and CA3 spines (Nusser et al., 1998). In the somatosensory cortex of the rat, AMPA receptor concentration is similar in synapses of different sizes; thus, the larger the synapse the higher the actual number of AMPA receptors (Kharazia and Weinberg, 1999), while NMDA receptors are found at a higher concentration in smaller synapses. In any case, it is obvious that the distribution of different types of receptors among different types of synapses is a complex issue (Hadzic et al., 2017), so the relationship between synaptic size and receptor number is not simple and requires further research.

Different synaptic sizes have been associated with different functions. For example, it has been proposed that small dendritic spines are preferential sites for long-term potentiation induction, whereas large spines might represent physical traces of long-term memory (Matsuzaki et al., 2004; Kasai et al., 2010). Our data show that synaptic size follows a log-normal distribution, which is unimodal and continuous, so neither AS nor SS can be divided into two groups on the basis of synaptic junction size. Therefore, if the function of “learning or memory” synapse depends on synaptic size, there would not be a clear-cut transition between the two types of synapses. Additionally, it has been proposed that the functional role of synapses may also depend on the sharp decrease of event-to-event variability as synaptic size grows, so the functional transition between learning and memory synapses would be faster than if it depended on synaptic size alone (Montes et al., 2015). In any case, if synapses of different sizes serve different functions, synapses on dendritic shafts must also be taken into account. Although such synapses are not the predominant type (∼15% of AS and 73% of SS; see also Santuy et al., 2018), their mean sizes are larger than axospinous synapses, both for AS and SS.

### Horseshoe-shaped and perforated synapses

Synaptic junctions with deep indentations (horseshoe synapses) and perforated synapses were scarce in our sample; even if we pool together horseshoe-shaped and perforated synapses, they only accounted for ∼7% of the whole population. The question arises about whether they are a separate population of synapses, with different morpho-functional features from the predominant macular synapses. These types of synapses are mainly located in the right tail of the synaptic size distributions, so their mean area is larger than the mean area of macular synapses, in line with numerous studies (Calverley and Jones, 1987; Geinisman et al., 1987; Jones and Calverley, 1991; Harris et al., 1992). Nevertheless, the sizes of horseshoe-shaped and perforated synapses also greatly overlap with the sizes of macular synapses, so there is not a boundary separating them from macular synapses (Fig. 6). Regarding the perimeter of horseshoe and perforated synapses, again we did not find any boundary, since the perimeter tends to be more complex as the PSD gets larger, regardless of the presence of perforations.

If we interpret perforations and deep indentations as dynamic, nonpermanent, features that may only depend on the molecular turnover of the constituents of the PSD, then perforated and horseshoe PSDs would belong to the same pathway as macular PSDs. The smallest synapses would have a macular shape whose perimeter would get progressively more tortuous as they grow. Deep indentations and perforations would appear (and eventually disappear) as the PSD becomes larger. The incorporation of receptors into the PSD depends on lateral diffusion from the surrounding plasma membrane (Choquet and Triller, 2013; Li and Blanpied, 2016) and on processes of endocytosis and exocytosis from the endosomal compartment (Choquet and Triller, 2013; Kneussel and Hausrat, 2016). In this scenario, we can hypothesize that indentations and perforations could be the morphologic correlate of a more active, or just more apparent, turnover of receptors in larger PSDs. In fact, it has been shown in the hippocampus that the relative proportions of horseshoe, perforated, and fragmented or partitioned synapses (synapses that have several irregular small disk-shaped PSDs, with no connection between them) do change after the induction of long-term potentiation (Geinisman et al., 1993; Toni et al., 2001). This phenomenon may or may not take place in the neocortex, where we have found horseshoe and perforated PSDs but not partitioned synapses. The fact that very different types of synapses such as AS and SS have perforations also suggests that these perforations are the result of a general, nonspecific mechanism, related to synaptic growth and remodeling.

Alternatively, perforated and probably also horseshoe-shaped synaptic junctions may belong to different populations of synapses. The main argument favoring this hypothesis is that in certain terminals, specifically in thalamocortical boutons in Layer IV, perforated synapses are frequent (Bopp et al., 2017; Rodriguez-Moreno et al., 2017), while they are scarce if we consider the whole synaptic population. Our data seems to contradict this hypothesis mainly because the proportion of perforated synapses is very similar in Layers II–VI. However, thalamocortical synapses represent only a minor proportion of Layer IV synapses (<10%; da Costa and Martin, 2009) and, therefore, their number may not be high enough to contribute to a significant difference with other layers. Species and age differences must also be taken into consideration, since the proportion of perforated synapses in Layer II/III of the visual and frontal cortices of the adult mouse seem to be larger than those reported here (Hsu et al., 2017).

### Curvature of the SAS

The relevance of the curvature of the synapse has been discussed since the seventies when Jones and Devon (1978) described changes in the curvature when administering anesthetics. Diverse studies led to the conclusion that positively curved synapses represented functional synapses, while negatively curved synapses were nonfunctional. Later studies revoked this view, as they showed that many other factors could influence the curvature of synapses (for example, the region studied; positively curved synapses predominated in the cortex while negatively curved synapses predominated in the hippocampus; Calverley and Jones, 1990). Nevertheless, more recent studies suggest that changes in the synaptic curvature may influence synaptic efficacy (Medvedev et al., 2010). In the present study, we found that SAS curvature was larger for SS than for AS in all layers. Furthermore, for AS, statistically significant differences were found between all layers except between Layers III and V. Curvature differences between SS were found between Layer IV and all the other layers, and this was also the case for Layer VI. Therefore, if synaptic curvature has an influence on synaptic efficacy, our results would indicate that this characteristic is layer and synaptic-type dependent. However, there was no correlation between SAS area and curvature for AS or SS. Since the area of the SAS seems to be related to the strength of synapses, the significance of the differences in synaptic curvature found between different layers and types of synapses observed in the present study remains to be determined.

### Concluding remarks

Collectively, the results indicate that there are laminar-specific similarities and differences regarding the size and shape of synaptic junctions. The functional implication of these variations is unknown but they may be related to synaptic attributes of particular synaptic circuits which are characteristic of each layer. The data obtained in the present study is based on the analysis of thousands of 3D-segmented synaptic junctions, providing a robust set of morphologic data. Since currently-available 3D quantitative data are rather scarce and mainly based on individual cases, the present results in conjunction with other crucial microanatomical data, such as the number and distribution of different types of synapses and the identification of postsynaptic targets in different cortical layers, will help to better understand the structure of microcircuits and to build realistic cortical models.

## Synthesis

Reviewing Editor: Alfonso Represa, INSERM Decisions are customarily a result of the Reviewing Editor and the peer reviewers coming together and discussing their recommendations until a consensus is reached. When revisions are invited, a fact-based synthesis statement explaining their decision and outlining what is needed to prepare a revision will be listed below. The following reviewer(s) agreed to reveal their identity: Shawn Mikula.

In this manuscript, the authors use ion bean milling and scanning electron microscopy (FIB-SEM) to investigate the size and shape of symmetric and asymmetric synapses in the somatosensory cortex of juvenile Wistar rats. They developped a new method that extracted the synaptic apposition surface from synaptic junctions in 3D, from up to 6891 synapses with complete synaptic junctions, this allowing the establishment of comparisons in different cortical layers. They demonstrate that there are laminar-specific similarities and differences in the size and shape of cortical symmetric and asymmetric synapses. The report is well written and illustrated; the statistical analyses performed are appropriate and support authors conclusions. The main limitation of this study is the failure to differentiate between neurochemical sub-classes in SS and AS synapses, but there are methodological limitations to this issue and it does not detract from the strength of the paper. The discussion is well balanced and interesting and highlight the interest of the research performed and the limitation of the interpretation in terms of functional implications. Data presented will be helpful for people investigating at the structural level cortical networks.
